# Healthy Lifestyle: Relationship between Mediterranean Diet, Body Composition and Physical Fitness in 13 to 16-Years Old Icelandic Students

**DOI:** 10.3390/ijerph15122632

**Published:** 2018-11-24

**Authors:** Pablo Galan-Lopez, Francis Ries, Thordis Gisladottir, Raúl Domínguez, Antonio J. Sánchez-Oliver

**Affiliations:** 1 Faculty of Educational Sciences, University of Seville, 41013 Sevilla, Spain; pgalan1@us.es (P.G.-L.); fries@us.es (F.R.); 2 Research Center for Sport and Health Sciences, School of Education, University of Iceland, 105 Reykjavík, Iceland; thg@hi.is; 3 Faculty of Health Sciences of Universidad Isabel I, Universidad Isabel I, 09004 Burgos, Spain; raul.dominguez@ui1.es; 4 Faculty of Sports Sciences of Universidad Pablo de Olavide, Universidad Pablo de Olavide, 41013 Sevilla, Spain; 5 Human Motricity and Sports Performance Area, University of Seville, 41013 Sevilla, Spain

**Keywords:** adolescents, physical fitness, Mediterranean diet, body composition

## Abstract

Childhood and adolescent obesity are currently among the greatest challenges for public health. Physical activity, physical fitness, and adherence to the Mediterranean diet (MD), representing powerful indicators of healthy lifestyles, are shown as determinant factors in the prevention and treatment of obesity. The aim of the present study has been to analyse the relationship between health-related physical fitness components, body composition, and adherence to MD in 387 Icelandic adolescents of 13–16-years old (54% boys). The ALPHA Fitness Test was used to measure physical fitness and body composition. The KIDMED questionnaire was used to assess the adherence to MD among participants. Associations between variables were tested according to gender and age using linear regression models and analysis of variance. Participants with high/medium adherence to MD showed significantly higher endurance scores in both the boys and the girls. Gender differences were found. The boys in high/medium MD categories had significantly lower fat percentages and ran a 4 × 10 m sprint faster than the girls. The girls scored higher than the boys in endurance and speed-agility tests. It can be concluded that a high and medium adherence to MD is associated with high and very high endurance in both the girls and the boys.

## 1. Introduction

Noncommunicable diseases, cardiovascular diseases, cancer, respiratory diseases, obesity, and diabetes are the main causes of death worldwide [1]. These deaths are caused, in large part, by poor diet, physical inactivity, and the consumption of alcohol and tobacco [2,3], thus establishing lifestyle as a good predictor of health and morbidity and mortality [4,5].

Childhood and adolescence are important periods of life, since many physiological and psychological transformations take place at these ages. Similarly, lifestyle and healthy or unhealthy behaviours are established during these years, which may influence adult behaviour and health status [6,7]. In children and young people, the prevalence of overweight and obesity has increased in recent years [8], causing premature deaths and increasing the risk of cardiovascular and metabolic diseases [9]. Childhood and adolescent obesity are a transcendental challenge for public health, both in its magnitude and in its consequences [10]. Lifestyle intervention is the most common treatment strategy in children and adolescents with obesity. Although there are studies with significant effects of lifestyle treatment in children and adolescents with obesity [11], additional research is required to draw conclusions about this type of population [12,13].

Among the habits that lead to a healthy lifestyle are physical activity (PA) and eating healthily [14]. Defined as ‘any bodily movement produced by skeletal muscles that results in energy expenditure’ [15], PA is a vital part of a healthy lifestyle and has been extensively documented and associated with health benefits in children and adolescents. Some of the benefits include reductions in blood cholesterol, hypertension, metabolic syndrome, obesity, and associated health problems such as diabetes mellitus type 2, cardiovascular diseases, or bone health problems in this population [16,17,18,19,20,21,22]. Moreover, physical fitness, mainly cardiorespiratory fitness, muscular fitness, and motor ability, have been shown to be powerful markers of health in young people [23]. Several PA tracking studies have been analysing activity patterns in childhood and adolescence and the risk of maintaining sedentary behaviours [24]. A low level of PA is associated with metabolic risk factors in young people that can also persist until adulthood [25].

Food intake in adolescence is a significant predictor of intake in adulthood [26]. In the context of overall dietary patterns, the MD has been accepted as one of the healthiest dietary patterns in the world [27], showing significant protection concerning mortality and morbidity when there is high adherence to it [28,29,30,31]. The MD has shown health benefits by reducing cardiovascular diseases, type 2 diabetes, certain types of cancer, and some neurodegenerative diseases [27,28]. Studies focusing on the influence of the MD in children and adolescents have increased in recent years [32]. In general, adherence to the MD has been shown to be associated with physical benefits and high levels of health-related quality of life, reducing the different factors associated with obesity, among others [33,34].

There is growing evidence that health behaviours are grouped. For example, the combination of regular PA and healthy eating habits helps to maintain and improve health and physical and mental well-being [35]. During youth, healthy eating combined with regular PA increases the likelihood of a healthy pattern of consistent physical maturation [36]. In addition, there are independent and combined associations between physical fitness, physical activity, and adherence to the MD with quality of life related to health in children, adolescents, and adults [33,37,38,39,40], with significant improvements in joint interventions [41,42,43].

However, despite all the benefits mentioned above, the current data show unhealthy patterns of eating and PA during the transition from childhood to adolescence [44], substantially contributing to the global burden of morbidity, mortality, and disability [45], and increasing the prevalence of overweight and obesity at those ages [46]. As mentioned before, obesity and hypertension, among others, have been largely attributed to unhealthy diets and a decrease in PA [47]. Thus, PA and nutrition are shown as fundamental pillars in the prevention and control of obesity [48,49].

The aim of the present study is to analyse the independent associations between health-related physical fitness components, body composition, and adherence to the MD of adolescents from Reykjavik (Iceland).

## 2. Materials and Methods

### 2.1. Study Sample and Design

The study design was cross sectional, including 13–16-year-old students from the capital area of Reykjavik, Iceland. A total of 439 participants (235 boys and 204 girls) were selected for the present research. Finally, 387 adolescents (209 boys (54%), Mage = 13.57, SD = 1.13 and 178 girls (46%), Mage = 13.38, SD = 1.14) took part in it, which yields a participation rate of 88.15%.

The subjects were recruited from two different schools in Reykjavik: Seljaskóli School (54.2%) and Ölduselsskóli School (45.8%). The inclusion criteria for this research were: male and female participants between 13 and 16 years old who had submitted the signed informed consent by their parents/guardians. Related to their health status, participants in the present investigation were those subjects who, due to their state of health, could participate regularly in the subject of Physical Education. The participants did not have any type of cognitive or physical/motor limitations The National Committee of Bioethics of Iceland approved the present study (Ref.: VSNb2017030026/03.01), which ensured that all the procedures related to research involving human beings would be carried out in complete safety. Written informed consent was obtained from the parents of the participants. The adolescents were asked for verbal consent, while they were informed that participation was voluntary and that they could leave the study at any time.

### 2.2. Instruments

Adherence to a Mediterranean Diet Questionnaire (KIDMED): The KIDMED questionnaire, previously validated, was used to evaluate the adherence to MD in adolescents (http://www.aulamedica.es/nh/pdf/9828.pdf). The questionnaire consists of 16 items, where 12 questions assume a positive score for adherence to MD (consumption of yogurt and dairy products, consumption of legumes, use of olive oil, consumption of vegetables, fruits, fish, cereals, rice, pasta, and nuts) and four questions assume a negative score (consumption of fast food, not having breakfast daily, consuming sweets several times a day, consuming industrial pastries). Affirmative answers to questions that represent a negative connotation in relation to the MD are worth −1 point and affirmative answers to questions that represent a positive aspect in relation to the MD are worth +1 point. Negative answers do not score [50,51,52].

Therefore, this index can range from 0 (minimum adherence) to 12 (maximum adherence). The sum of the values of this questionnaire gives rise to the KIDMED index, which is classified into three categories: From 8 to 12: an optimal MD (high adherence); from 4 to 7: a need to improve the food pattern to adapt it to the Mediterranean model (average adherence); and from 0 to 3: a very low-quality diet (low adherence) [50,51,52].

Alpha Fitness test battery: Physical fitness and anthropometric variables were assessed by a modified version of the extended ALPHA fitness test battery, (Ref: 2006120)). Skin folds were omitted for limited time reasons and the 4 × 10 m speed-agility test was added to the version in order to have more information about physical fitness. The protocol marked on the ALPHA-Fitness Battery for measurement was followed at all times [53].

Body composition: The height of the subjects was recorded barefoot with an accuracy of 0.1 cm using a portable stadiometer (Seca 213, Seca, Hamburg, Germany). The weight of the participants was measured with an accuracy of 0.10 kg, the subjects wore light clothing, and a portable electronic scale was used. Body fat percentage (BF%) was measured by bioelectrical impedance (Tanita Inner Scan BC-543, Tanita, Tokyo, Japan). Body mass index (BMI) was calculated from the ratio of body weight (kg) to body height (m^2^). Waist circumference was measured with a non-flexible measurement tape (Seca 201, Seca, Hamburg, Germany) with the adolescent standing upright and with an accuracy of 0.1 cm. The measuring point was the narrowest part of the space between the lowest rib and the anterior superior iliac spine at the end of normal expiration.

Cardiovascular fitness was assessed with the multistage 20 m shuttle run test (Leger et al., 1988) [54]. In this test, the participants had to run a distance of 20 m, adjusting their speed to the rhythm of the audio signals that were emitted from a previously recorded CD. The subjects finished the test when they could not reach the line a second time concurrent with the audio, or when the subject stopped due to fatigue. The initial speed was 8.5 km/h, with this being increased by 0.5 km/h per minute [55].

Lower body explosive muscle strength was assessed using a standing long jump. The participants, placed behind the jumping line with their feet together, pushed hard and jumped as far away as possible, contacting the ground with both feet simultaneously and in a vertical position. The distance was measured from the rearmost heel to the jumping line and was always performed on a non-slippery surface.

Upper body maximal muscle strength was measured by means of handgrip strength using a hand dynamometer with an adjustable grip (TKK 5401 Grip D, Takey, Tokyo, Japan). The examiner showed the correct way of execution and adjusted the grip measure according to the size of the hand [56]. The test was performed twice, and the best result was recorded, calculating the average of the two hands. The subjects were verbally encouraged to “squeeze as hard as possible” and to exert the maximum effort for at least two seconds (s). Speed-agility was tested using the 4 × 10 shuttle run test. The examiner showed the correct way of execution. The test was performed twice, and the best result was recorded (s). The participants had to run, as fast as possible, the distance between the two lines placed 10 m away, change a series of sponges (three times), and run back to the starting line.

### 2.3. Methodology

All participants performed the test battery and the KIDMED questionnaire during the time corresponding to their physical education classes. The different tests were organised as a circuit and the participants carried out all the tests consecutively, except for the cardiovascular fitness test, which was performed by several students at the same time and on a different day. The development and performance of all physical tests lasted one hour for each class of 20–25 students.

### 2.4. Data Analysis

Quantitative variables were presented as means (M) and standard deviations (SD), while frequencies and percentages (%) were used for qualitative variables. After verifying the normality of the variables by means of the Kolmogorov-Smirnoff test, a Student-T test for independent samples was used to perform a comparative analysis of the quantitative variables of body composition and physical fitness between the boys and the girls. In addition, to check for possible differences in the proportion of subjects that are in the different categories in the % of body fat for an adolescent population established by Moreno et al. (2006) [57], a Chi-square test was performed to check for the possible differences between genders. In order to analyse the degree of adherence to an MD (low, medium or high), both in the boys and the girls, in relation to the different variables of body composition and physical fitness, after checking homoskedasticity by means of Levene’s test, a one-way ANOVA and, in the case of statistically significant differences, a Bonferroni post-hoc test, was performed. Furthermore, a Chi-square test was performed to check for the possible differences in each of the questions that compose the KIDMED questionnaire grounded on the normative levels of body fat % established by Moreno et al. (2006) [57]. The level of statistical significance was set as *p* < 0.05. All statistical analyses were carried out using the SPSS statistical package (version 18.0, SPSS Inc., Chicago, IL, USA).

## 3. Results

The characteristics of the participants, including age, body composition, and physical fitness, are shown in Table 1. Although anthropometric data show a statistically higher weight (+7.5%, *p* = 0.010) and height (+3.7%, *p* < 0.001) in the boys, there were no statistically significant differences in relation to BMI (*p* = 0.241) between the boys and the girls. In addition, the boys showed a lower percentage of body fat (−33.4%, *p* < 0.001), but a significantly higher waist circumference (+5.8%, *p* < 0.001).

**Figure 1:**
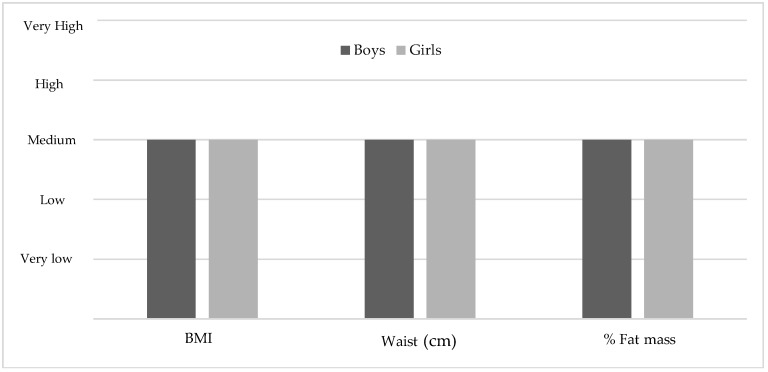
Classification of the mean levels of body composition variables based on the average levels established by Moreno et al. (2006, 2007) [57,58].

However, by relativising the average data of the boys and the girls to the different levels of body composition established by Moreno et al. (2006, 2007) [57,58], it is confirmed that the values of BMI, waist and % of body fat are classified as ‘medium’ in each of the parameters (see Figure 1).

**Figure 2:**
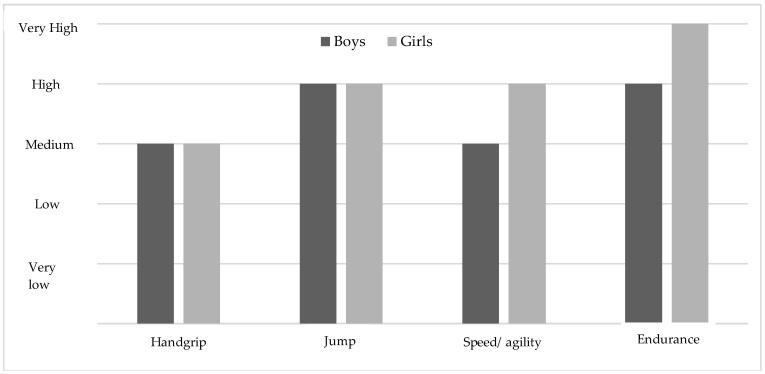
Mean levels of physical fitness variables based on the average levels established by Ortega et al. (2011) [59].

In relation to the physical fitness components, the boys showed significantly higher performances in each of the tests (*p* < 0.001). However, when categorising the average levels of the boys and the girls to the different levels of physical fitness established by Ortega et al. (2011) [59], the average value of the boys is established as an average value and the girls show high levels. In endurance, the boys show a high level and the girls very high (see Figure 2).

**Figure 3:**
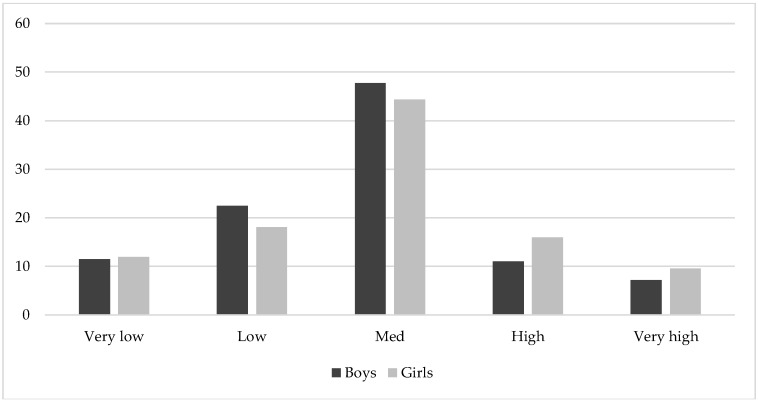
Proportion of boys and girls in each of the Alpha Fitness categories based on the percentage of body fat.

In addition, Table 1 highlights no differences in the KIDMED-index when comparing both the boys and the girls. Analysing the participants in the different categories established based on the percentage of body fat %, statistically significant differences were observed between both (*p* = 0.003), where more girls showed high and very high values and a lower % ranked in low and medium (see Figure 3).

**Figure 4:**
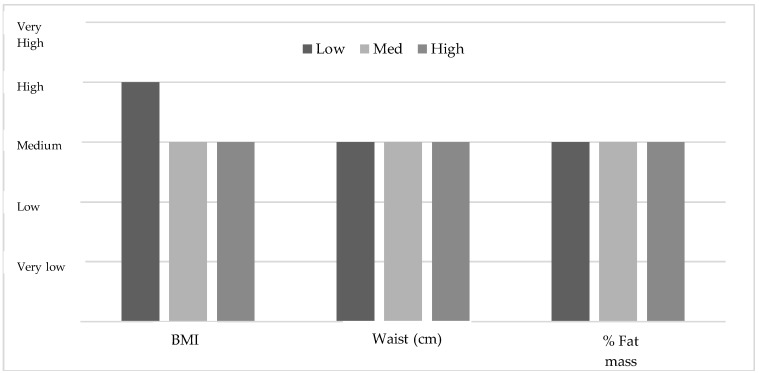
Mean levels of body composition variables in the boys based on the grouping by Moreno et al. (2006, 2007) [57,58].

**Figure 5:**
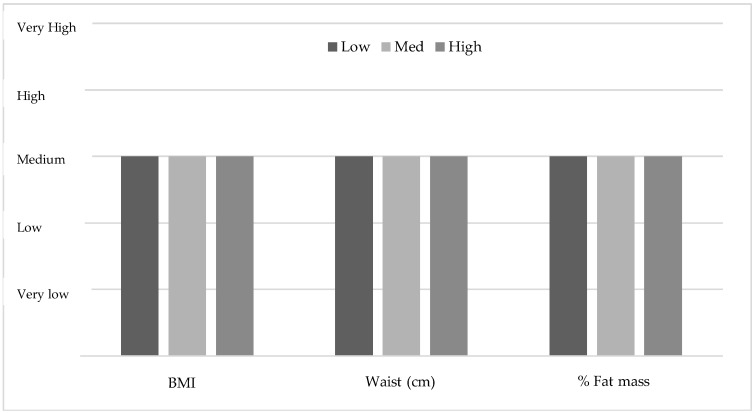
Mean levels of body composition variables in the girls based on the grouping by Moreno et al. (2006, 2007) [57,58].

When performing a stratification of the sample based on the degree of adherence to MD, 14.99% showed low adherence, 60.72% an average level, and 24.29% a high level. When comparing body composition in relation to the degree of adherence to MD, it was found that, although there were no statistically significant differences in girls, boys showed a higher % of body fat among those who had a low adherence (21.84%) in comparison to a medium (16.79%) or a high adherence (16.21%) (*p* = 0.006) (see Table 2). When categorising the average levels with the classification established by Moreno et al. (2006, 2007) [57,58], the boys and the girls with different levels of adherence to MD present a medium level in the % of body fat and waist circumference (see Figure 4 and Figure 5). On the contrary, when comparing the normative levels of the mean levels of these variables with the categorisation established by Moreno et al. (2006, 2007), it is verified that both genders present average levels in waist circumference and body fat percentage [57,58]. However, in the BMI variable in the boys, even though there were no differences (*p* > 0.05), the average level of subjects with low adherence to MD is rated as a high level, whereas those who show a medium or high adherence present an average level.

**Figure 6:**
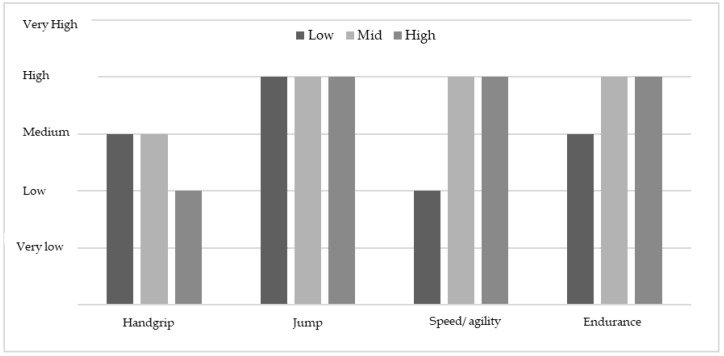
Mean levels of the physical fitness variables in the boys based on the average levels established by Ortega et al. (2011) [59].

**Figure 7:**
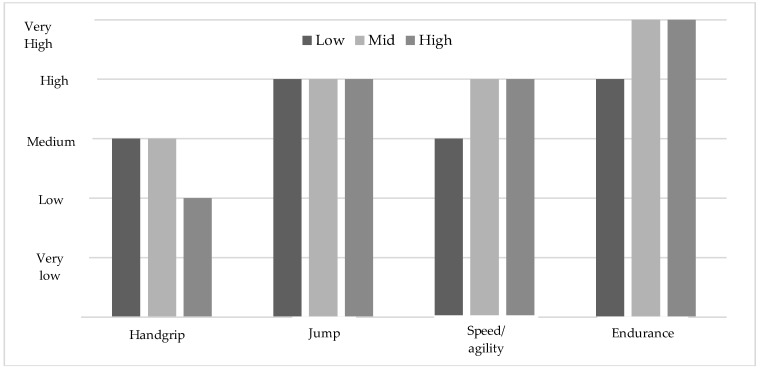
Mean levels of the physical fitness variables in the girls based on the average levels established by Ortega et al. (2011) [59].

The results of the fitness tests showed a higher performance in endurance, both for the boys and the girls, among the participants with a medium or high adherence to MD than those with low adherence (*p* < 0.05) (see Table 2). Moreover, when comparing the endurance classification of the average levels in the boys with a low adherence with the normative levels established by Ortega et al. (2011), these are medium [59], while those that have a medium or high adherence are high (see Figure 6 and Figure 7).

Furthermore, the boys with a high and medium score in the MD have a significantly lower fat percentage and run faster on the 4 ×1 0 m sprint test in comparison to those with a low adherence to MD (*p* = 0.002), with no such difference being found in the girls. However, the average levels of the girls with a low level of adherence to MD were medium, whereas the girls with a medium or a high adherence were classified as high according to Ortega et al. (2011) (ALPHA fitness test) [59]. Regarding the jump test, no statistically significant differences were found in relation to adherence to MD. The same happened with the hand grip test, although the average levels of manual grip strength are classified as low in the boys and the girls with a high adherence to MD compared to the medium levels of participants with a low and medium adherence to MD.

## 4. Discussion

The present study is the first to analyse and describe the health-related physical fitness together with the adherence to MD of Icelandic adolescents.

As expected after analysing the body composition of the participants, significant differences were obtained between the boys and the girls in weight, height, % of body fat, and waist circumference (see Table 1). These results are similar to those obtained in several studies on the adolescent population [60,61,62,63], in which girls had higher levels of adiposity, whereas boys had higher weight, height, and waist circumference values. Both the boys and the girls show average values of BMI, % of body fat, and waist circumference (see Figure 1).

The weights, heights, and BMIs of the present study sample are comparable to the reference values provided by Ortega et al. (2011) [59]. Unlike what was found by Wärnberg et al. (2006) [64], there is no prevalence of obesity in the adolescent participants of this study as the BMI, waist circumference, and % of body fat are considered medium values (see Figure 3).

In relation to the performance in the physical fitness tests, the boys obtained significantly better results in manual dynamometry, the long jump, the 4 × 10 m sprint, and the endurance test (see Table 1). These results are similar to those of previous studies carried out [55,65,66,67]. However, when analysing the mean values of the different tests in relation to Alpha Fitness categories, the girls show the highest scores (see Figure 2). These results differ from several studies where boys score higher on these tests [66,68]. It is possible that three hours of mandatory PE classes and swimming lessons in secondary education may contribute to obtaining these results [69].

As mentioned before, MD is considered one of the healthiest dietary patterns [70], with benefits on a physical and mental level, among others [71,72,73]. The results of the present study show a low/poor MD adherence (14.99%) and are superior in an average (60.72%) and high adherence (24.29%). In addition, no significant differences were observed in the adherence to the diet according to the gender of the participants. These results are also superior to those of studies conducted in Mediterranean countries [74,75,76]. These results are different with respect to a recent study that analysed, in a similar way, the adherence to MD in non-Mediterranean countries, showing worse final results than ours with a poor (39%), medium (47.7%), and high (13.3%) adherence [73].

Several recent articles directly associate the adherence to MD with the weight and the BMI of the participants [77,78]. These data are similar to those obtained here, as the participants with the highest adherence to MD are those who also show regular weights and BMIs. Those with a low adherence to MD show a high BMI, although this is not significant. It should be noted that these values are significant when considering fat % (see Table 2), so it would be substantial to see which variable, BMI or fat %, has more importance in the three subgroups (low, medium, and high) that result from a better adherence to the MD (see Figure 4 and Figure 5).

In contrast to the results obtained by Ozen et al. (2015) [79], which showed important differences in relation to a high and low adherence in the population analysed, with a clear tendency to abandon the MD, the results found in the present study display a tendency to maintain or even increase the MD patterns related to this type of diet, since a medium and high adherence gather 85% of the participants (see Figure 4 and Figure 5).

These results from the KIDMED index are in line with the results obtained in other studies [77]. In addition, the results found are similar to studies carried out in southern European countries, where a large part of the sample is at a medium level of adherence to MD [80,81].

Regarding the relationship of the MD with the waist circumference, Bacopoulou et al. (2017), after studying more than 1600 subjects of a similar age to those of the present research, determined that the increase in adherence to MD was associated with a decrease in the perimeter of the waist, indicating a potential for school interventions to fight against abdominal obesity in adolescents [71]. This matches with the results of the present study and with the findings of Schröder et al. (2010), where more than 60% of the participating subjects presented a medium adherence to MD and medium, low, and very low waist circumference values [38].

As a novel aspect, the present study searched for relationships between health-related physical fitness, adherence to MD, and body composition (see Table 2 and Figure 6 and Figure 7) in Icelandic adolescents. Significant differences were found in the tests of 4 × 10 m and endurance in the boys, and endurance in the girls with respect to those participants that show low adherence, compared with those with a medium or high adherence to MD. The disparity between genders in performance scores can be explained in the different processes of the adolescents’ development. Girls experience development earlier than boys, which determines their ability to develop higher levels of strength, speed, and endurance [82].

The results mentioned in the previous paragraph are consistent with the conclusions of recent research. Muros et al. (2017), for example, found a positive relation between a high performance in the resistance test and a high adherence to MD [77]. Evaristo et al. (2018) not only demonstrated the relationship between a high adherence to MD and high levels of health-related physical fitness, but the subjects also showed high levels of health-related quality of life [70].

Despite the strength of the study, it is also important to acknowledge the limitations of the current research, which may restrict the generalisability of our findings and possible alternative interpretations. First, our data are cross-sectional and, therefore, do not enable us to infer the causal direction of our predictions. Nevertheless, they can be used as valuable indications to be considered for future research. Second, some of the data collected (KIDMED) were self-reported, which could lead to an error in the reports and recall bias due to the nature of the study. In addition, it must be borne in mind that the KIDMED questionnaire, although it was used to observe the adherence to MD does not contemplate the content and intake of nutrients consumed by the sample, which may be a confounding factor to be taken into account.

## 5. Conclusions

The adolescents participating in this study show medium/high levels of health-related physical fitness, with the girls obtaining slightly higher results. The participants’ adherence to MD is classified as medium/high since 60% of the participants are in the middle level and almost 25% are in the high level.

The results found showed significant correlations between MD and the endurance test in the girls and the boys. A high adherence to MD also correlates with better results in endurance and agility speed tests in the boys.

This research shows the importance of developing and maintaining an adequate physical fitness and, together with a medium or high level of adherence to MD, it culminates in a better health-related quality of life in adolescents. Both the boys and the girls that showed a medium and high adherence to MD had the highest scores in the health-related physical fitness tests [50].

These results agree with those obtained in the Spanish and Portuguese adolescent population, since a high adherence to MD is related to higher levels of perceived quality of life, within which a good level of physical fitness is found. Moreover, a high adherence to MD is associated with a significant improvement in physical health and with lower obesity, a fact that is consistent with recent results [42].

Finally, this research appeals for the development of public health programmes, awareness campaigns, and the creation of PA and healthy eating environments for children and adolescents [64]. Not only an adequate diet is sufficient [65], but a minimum of daily physical activity practice is necessary to avoid the appearance of diseases derived from a sedentary lifestyle [40] and consequently, a poor quality of life [66].

## Figures and Tables

**Table 1:** Anthropometric characteristics and physical fitness variables (*n* = 387).

Variables	Mean ± SD	Boys (*n* = 209)	Girls (*n* = 178)	*p*-Value
Age (year)	13.48 ± 1.14	13.57 ± 1.13	13.38 ± 1.14	0.097
Weight (kg)	57.29 ± 13.53	59.20 ± 14.21	55.05 ± 12.36	0.010 *
Height (m)	1.64 ± 0.10	1.67 ± 0.11	1.61 ± 0.81	<0.001 *
BMI (kg/m^2^)	22.26 ± 4.72	22.17 ± 4.91	22.37 ± 4.49	0.241
Body fat (%)	21.37 ± 8.79	17.37 ± 8.44	26.07 ± 6.61	<0.001 *
Waist (cm)	72.05 ± 10.12	73.91 ± 10.41	69.86 ± 9.34	<0.001 *
Handgrip (kg)	25.95 ± 7.01	28.18 ± 7.90	23.33 ± 4.58	<0.001 *
Jump (cm)	171.94 ± 1.51	182.94 ± 28.60	159.02 ± 25.51	<0.001 *
4 × 10 m (s)	11.80 ± 1.40	11.53 ± 1.59	12.11 ± 1.06	<0.001 *
Endurance (CRF)	6.73 ± 2.52	7.52 ± 2.62	5.80 ± 2.04	<0.001 *
KIDMED Index	5.83 ± 2.31	5.82 ± 2.18	5.84 ± 2.46	0.940

Note: SD = Standard Deviation, BMI = Body Mass Index, Waist = Waist Circumference, CRF = Cardiorespiratory Fitness. * Expresses statistically significant differences between boys and girls (*p* < 0.05).

**Table 2:** Anthropometric characteristics and physical fitness variables of the sample stratified according to adherence to MD in both boys and girls (Data presented as Mean ± SD).

Variables	Boys					Girls		
	Low (*n* = 58/387)	Med (*n* = 235/387)	High (*n* = 94/387)	*p*-Value	Low (*n* = 58/387)	Med (n = 235/387)	High (*n* = 94/387)	*p*-Value
Age (year)	13.37 ± 1.30	13.61 ± 1.10	13.58 ± 1.13	0.562	13.18 ± 0.98	13.29 ± 1.11	13.07 ± 1.11	0.678
Weight (kg)	61.15 ± 15.62	59.72 ± 14.51	56.80 ± 12.48	0.332	57.34 ± 9.90	55.31 ± 12.78	52.83 ± 12.67	0.308
High (m)	1.64 ± 0.10	1.67 ± 0.11	1.66 ± 0.11	0.338	1.62 ± 0.60	1.61 ± 0.09	1.60 ± 0.07	0.586
BMI (kg/m^2^)	23.90 ± 6.06	22.07 ± 4.71	21.41 ± 4.52	0.080	22.34 ± 5.01	22.52 ± 4.40	22.01 ± 4.43	0.824
Body fat (%)	21.84 ± 10.01 ^a^	16.79 ± 7.92	16.21 ± 8.03	0.006 *	26.50 ± 6.52	25.93 ± 6.69	26.16 ± 6.60	0.919
Waist (cm)	76.95 ± 11.75	73.87 ± 10.68	72.24 ± 8.56	0.142	70.48 ± 8.57	70.29 ± 9.54	68.33 ± 9.36	0.479
Handgrip average (kg)	27.99 ± 8.43	28.81 ± 7.88	26.75 ± 7.59	0.286	24.36 ± 3.69	23.40 ± 4.47	22.45 ± 5.31	0.225
Jump (cm)	177.2 ± 31.4	185.0 ± 27.7	181.3 ± 29.1	0.362	155.75 ± 25.68	160.02 ± 26.42	158.62 ± 23.33	0.730
4 × 10 m (s)	12.42 ± 2.00 ^a^	11.29 ± 1.21	11.59 ± 1.96	<0.002 *	12.46 ± 0.90	12.04 ± 1.16	12.07 ± 1.17	0.174
Endurance (CRF)	6.28 ± 2.64 ^a^	7.68 ± 2.43	7.85 ± 2.88	<0.018 *	4.63 ± 1.32 ^a^	6.04 ± 2.12	5.94 ± 1.99	0.004 *

Note: SD = Standard Deviation, BMI = Body Mass Index, Waist = Waist Circumference, CRF = Cardiorespiratory Fitness. * Expresses statistically significant differences between groups (*p* < 0.05). ^a^ Expresses statistically significant differences between Low and Med and High.
